# Bone density of the cervical, thoracic and lumbar spine measured using Hounsfield units of computed tomography – results of 4350 vertebras

**DOI:** 10.1186/s12891-024-07324-1

**Published:** 2024-03-06

**Authors:** George Simion, Niklas Eckardt, Bernhard W. Ullrich, Christian Senft, Falko Schwarz

**Affiliations:** 1 Department of Neurosurgery, Jena University Hospital, Friedrich Schiller University Jena, Jena, Germany; 2 Department of Radiology, Jena University Hospital, Friedrich Schiller University Jena, Jena, Germany; 3 Department of Orthopedics and Traumatology, Friedrich Schiller University Jena, Jena, Germany

**Keywords:** Osteoporosis, Fracture, Bone mineral density, Spine, Hounsfield units

## Abstract

**Introduction:**

The assessment of bone density has gained significance in recent years due to the aging population. Accurate assessment of bone density is crucial when deciding on the appropriate treatment plan for spinal stabilization surgery. The objective of this work was to determine the trabecular bone density values of the subaxial cervical, thoracic and lumbar spine using Hounsfield units.

**Material and methods:**

Data from 200 patients who underwent contrast-enhanced polytrauma computed tomography at a maximum care hospital over a two-year period were retrospectively analyzed. HUs were measured with an elliptical measurement field in three different locations within the vertebral body: below the upper plate, in the middle of the vertebral body, and above the base plate. The measured Hounsfield units were converted into bone density values using a validated formula.

**Results:**

The mean age of the patient collective was 47.05 years. Mean spinal bone density values decreased from cranial to caudal (C3: 231.79 mg/cm^3^; L5: 155.13 mg/cm^3^; *p* < 0.001), with the highest values in the upper cervical spine. Bone density values generally decreased with age in all spinal segments. There was a clear decrease in values after age 50 years (*p* < 0.001).

**Conclusions:**

In our study, bone density decreased from cranial to caudal with higher values in the cervical spine. These data from the individual spinal segments may be helpful to comprehensively evaluate the status of the spine and to design a better preoperative plan before instrumentation.

## Introduction

Given the increasing age of patients, the evaluation of bone density is now more important than ever. When considering therapy options for surgical stabilization of the spine, evaluating bone density is a critical factor to take into account. There are already several studies in the literature showing a significant correlation between the Hounsfield units of a computed tomography and the bone density measured by DXA (Dual Energy X-ray Absorptiometry) [1–7].

The assessment of bone density is an essential part of diagnosing osteoporosis. The importance of osteoporosis in a clinical context stems from the elevated risk of fractures associated with the condition, which has gained greater attention with the aging of the population. Therefore, assessing bone density is of utmost importance [8, 9]. Worldwide, osteoporosis causes more than 8.9 million fractures annually [10]. It is typical for the diagnosis of osteoporosis to be made only after an osteoporotic fracture has occurred. Osteoporotic fractures lead to increased morbidity and mortality [11]. Using data from imaging investigations taken for another clinical purpose can help diagnose osteoporosis and lead to the initiation of osteoporosis treatment before the clinical onset of fractures.

It is well-established that age is an independent factor contributing to the decline in bone density [12, 13]. In their study, Yu et al. found that women after the age of 49 experience a more pronounced decrease in spinal bone density compared to men [14].

When it comes to measuring bone density, DXA is recognized as the gold standard [15]. Although DXA is the gold standard for measuring bone density, it has several limitations, such as its inability to distinguish between cortical and cancellous bone, its inability to examine specific spinal segments in the cervical and thoracic spine, its inability to provide volumetric measurements, its limited availability in the inpatient setting and its high cost [16–18].

Several techniques can be employed to assess bone density from a clinical CT scan, such as simultaneous calibration, asynchronous calibration, internal calibration, or utilizing Hounsfield units (HU) directly [19–21]. Standard quantitative CT (QCT) typically utilizes simultaneous phantom-based calibration to determine bone density. With this approach, a phantom containing hydroxyapatite is placed beneath the patient, and bone density is calculated from the CT values. This method helps minimize differences in bone density measurement between different CT scanner models. In contrast to simultaneous phantom-based calibration, the asynchronous calibration method for measuring bone density does not necessitate the presence of a calibration phantom during CT scans. With this method, patient and phantom scans are done separately, and the calibration phantom may be scanned at regular intervals, such as once a week or once a month [19]. The internal density calibration method offers an alternative to using a calibration phantom for opportunistic CT screening. With this method, in-scan regions of interest (ROIs) in different body tissues, such as subcutaneous adipose tissue and blood, are used for calibration. Michalski et al. found that internal calibration is as effective as phantom-based calibration, as evidenced by their cadaveric analyses [22]. Another method for determining bone density is the direct measuring of Hounsfield Units (HU) of a CT scan. Unlike other methods, this technique doesn't require any calibration, which makes it easier to use. However, to obtain accurate results, the CT scanner used for this approach should be produced by the same manufacturer or ideally be the same scanner model [19, 23]. Measuring bone density by determining HU doesn't entail any extra cost or radiation exposure since a CT scan is typically already performed after a traumatic event or prior to spinal instrumentation. The use of HU as a mean of measuring bone density is generally accepted as reliable [3, 5–7, 24]. Buenger et al. found that there is a strong correlation between HU measurements obtained from CT scans and bone density measurements obtained from QCT. They proposed conversion formulas for calculating QCT values based on HU measurements: QCT value = 0.7 × HU + 17.8 for native CT scans, and QCT value = 0.71 × HU + 13.82 for contrast-enhanced CT scans [1, 3].

Using the direct measurement of Hounsfield units in CT we already examined the bone density of the second cervical vertebra. The transitional area from dens to corpus vertebra showed statistically significant lower bone density values compared to adjacent regions [25]. To our knowledge, no scientific study currently exists that examines bone density of the entire spine and compares it according to anatomical location, age, and sex.

The aim of this work was to assess the trabecular bone density values using Hounsfield units in the individual vertebral bodies of the subaxial cervical spine, thoracic spine and lumbar spine. Furthermore, it was to be examined whether differences in bone density exist between the individual vertebral segments in relation to sex and age.

## Material and methods

This study is a monocentric retrospective data analysis. Two hundred patients who received contrast-enhanced polytrauma CT scans (256-slice Multi Detector Ct Scanner GE Healthcare Revolution; slice thickness 0.625 mm; tube spectra 80–120 kV, tube current: Smart mA 100–755, voxel size: 1,25 mm, pitch: 0.922:1, rotation time: 0.5 s, detection coverage: 80 mm) in a period between 01/01/2020 and 31/06/2021 at a maximum care hospital were included in the study. Inclusion of patients in the study followed a chronological order that was determined by their examination dates. The data was anonymously collected by one physician in an Excel spreadsheet. Basic information (patient age, sex, examination date) and Hounsfield units and vertebral bone density from C3 to L5 were recorded. Bone density values were calculated using the formula of Buenger et. al (QCT value = 0.71 × HU + 13.82) [1]. Stratification of the data was done by sex and age group. The overall study design and conduct was approved by the local ethic committee (Reg. No.: 2020-2030).

Patients without age limitation who underwent contrast-enhanced polytrauma CT were included. The entire spine had to be visualized in the CT scan in at least sagittal and axial planes.

Exclusion criteria were pathologies such as fractures in more than three vertebrae, surgery with instrumentation of the spine, signs of osteochondrosis in more than three vertebrae, spondylodiscitis, pronounced scoliosis or kyphosis, and artifacts due to implanted materials or other causes. In patients who had fractures, osteochondrosis, or artifacts in no more than three vertebrae, the HU of the affected vertebral bodies were not recorded.

### Measurement of the Hounsfield units

**Fig. 1:**
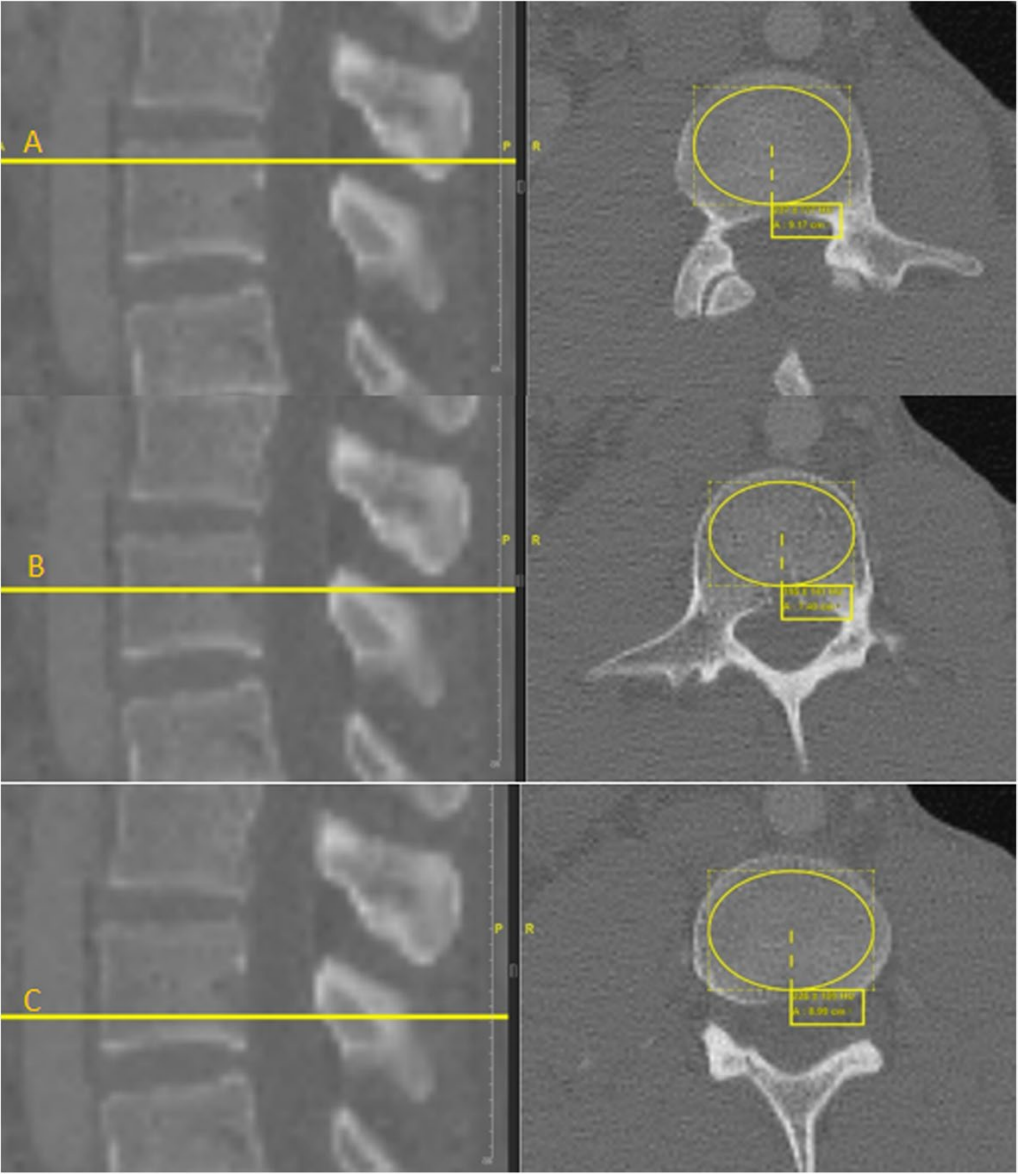
Determination of the HU of the L3 vertebral body in 3 localizations below the upper plate **A**, in the middle of the vertebral body **B** and above the base plate **C** using contrast-enhanced computed tomography

Hounsfield units (HU) were recorded in the axial plane. HU were measured with an elliptical measurement field in three different localizations within the vertebral body: below the upper plate, in the middle of the vertebral body excluding the Batson venous plexus and above the base plate. In the axial plane, the "region of interest" (ROI) was chosen as large as possible, leaving out the cortical bone (Fig. 1). In this way, the measurement was limited to only the trabecular bone [26].

The HU were measured in the Centricity Universal Viewer Zero (GE Healthcare, Chicago, USA). A mean value of the HU of the respective vertebral body was calculated from the above three values. The measurement of HU values was always performed by a single physician.

### Statistics

The data were recorded anonymously in Excel 2016 (Microsoft Corporation, USA). Statistical analysis was performed using SPSS 26 (IBM Inc, USA). Data was grouped by sex and age (10-year intervals).

Statistical analysis was carried out using nonparametric tests as the data did not have a normal distribution. Comparison of bone density of different localizations was performed using the two-sided Friedman test. Comparison of 2 localizations was performed using the two-sided Wilcoxon test. The sex-based comparison was performed using the two-sided Mann-Whitney U test. Age-by-age comparison was performed using the two-sided Kruskal-Wallis test. In order to compare each group with another, we employed multiple pairwise Mann-Whitney tests with Benferroni correction as the post-hoc test. For all analyses, a *p* value <0.05 was assumed to be significant.

## Results

A total of 200 patients were included (153 males and 47 females). The patients were on average 47.05 years old at the time of study (range: min. 10; max. 89).

**Fig. 2:**
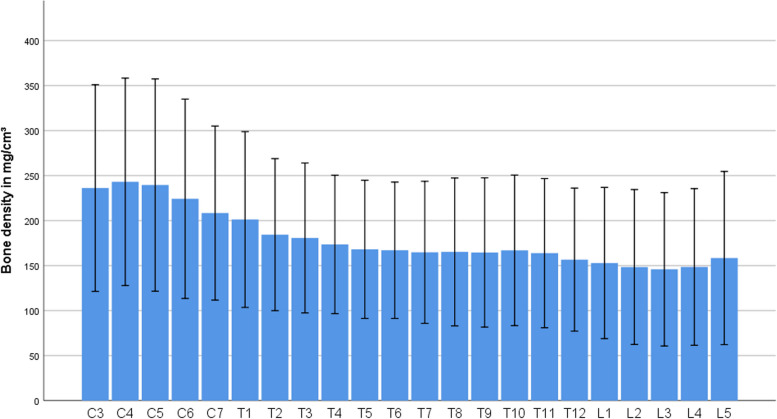
Mean values of bone density from vertebral bodies of all patients in mg/cm^3^. The error bars represent +/- 2 SD

The mean and median of bone density of all measured vertebral bodies was 181.53 mg/cm^3^ and 181.59 mg/cm^3^, respectively (min. 69.89; max. 337.50 mg/cm^3^). The bone density values of each vertebral body are presented in Table 1 and Fig. 2.

**Table 1:** Mean and median values of bone density from vertebral bodies of all patients in mg/cm^3^

Vertebral body	Mean	Median	Standard Deviation	Minimum	Maximum
C3	231.79	229.78	57.57	99.97	429,17
C4	239.53	237.94	57.91	103.04	456.39
C5	237.12	233.68	59.62	100.68	448.34
C6	222.28	220.90	55.17	104.70	441.95
C7	206.27	204.81	47.70	108.01	375.92
T1	198.10	198.18	49.66	62.57	357.93
T2	181.19	178.19	43.39	82.93	336.16
T3	177.66	177.36	42.70	84.82	340.42
T4	170.29	168.25	39.35	80.56	300.66
T5	165.45	163.87	39.50	66.60	309.89
T6	164.07	165.29	38.977	75.59	306.34
T7	161.85	162.92	40.230	66.36	288.59
T8	161.83	162.45	41.452	63.52	305.16
T9	161.84	164.81	41.985	31.57	299.71
T10	164.46	165.29	42.373	34.17	300.90
T11	161.32	161.50	42.420	65.29	298.53
T12	154.11	155.11	41.224	62.57	291.19
L1	152.03	156.53	42.821	55.00	298.77
L2	146.45	150.14	44.903	30.15	296.40
L3	143.45	146.35	43.832	51.81	298.29
L4	146.43	148.25	44.863	28.73	281.49
L5	155.13	157.71	49.513	27.31	314.62

*C* Cervical, *T* Thoracic, *L* Lumbar

After conducting the Shapiro-Wilk test (*p*<0.05) and visually inspecting histograms and QQ charts, it was determined that the bone density values for the cervical and lumbar spine, as well as most of the other data, did not follow a normal distribution. Thus, non-parametric tests were used for further statistical analysis.

There was a statistically significant difference between the bone density values of the cervical, thoracic and lumbar spine (*p*<0.001). The bone density decreased statistically significantly from cranial to caudal: the median bone density values of cervical spine were higher than the bone density values of thoracic spine (228.67/IQR 190-263 vs. 169.31/IQR 136-197 mg/cm^3^; *p*<0.001), and the median bone density values of thoracic spine were higher than the bone density values of lumbar spine (169.31/IQR 136-197 vs. 149.69/IQR 111-181 mg/cm^3^; *p*<0.001). In the lower cervical spine (C6 and C7), the values were statistically significantly lower than the values of the upper cervical spine (*p*<0.001). In the thoracic spine region, the same descending tendency from cranial to caudal was noted, with statistically higher values in the upper thoracic spine region (T1-T4) compared with the rest of the thoracic spine. In the lumbar spine, values in the L1 and L5 regions were higher than the other values (*p*< 0.001). There was no significant difference between the bone density values of L2, L3 and L4.

The difference between the bone density values of the whole spine between females and males was not statistically significant (169.16/IQR 137-223 vs. 185.94/IQR 152-208 mg/cm^3^; *p*=0.439). The difference between bone density values of individual spinal segments (cervical, thoracic, lumbar spine) between females and males was also not statistically significant (*p*= 0.449, *p*= 0.518, and *p*= 0.413, respectively).

There was a statistically significant difference between the bone density values of the different age groups (*p*< 0.001) (Table 2).

**Table 2:** Values of bone density from all the vertebral bodies of cervical, thoracic and lumbal spine of each age group in mg/cm^3^

Age	Location	N	Median	Mean	Std. deviation	Range
10-19	C	17.00	265.16	267.47	45.28	195.84
	T	17.00	201.28	211.00	36.31	154.89
	L	17.00	189.62	198.61	33.50	151.02
20-29	C	25.00	235.50	244.66	37.32	146.34
	T	25.00	187.08	194.18	27.18	108.62
	L	25.00	181.19	187.07	23.58	94.52
30-39	C	36.00	248.35	255.72	50.83	223.14
	T	36.00	195.26	192.84	30.29	129.14
	L	36.00	177.21	179.22	28.76	123.22
40-49	C	25.00	241.10	254.97	48.10	187.36
	T	25.00	181.68	183.43	25.87	103.00
	L	25.00	158.00	161.31	23.49	80.10
50-59	C	39.00	213.68	216.86	53.32	297.61
	T	39.00	145.72	148.39	27.89	145.38
	L	39.00	112.65	122.30	29.10	138.49
60-69	C	28.00	193.17	203.15	43.60	166.17
	T	28.00	144.65	147.16	29.07	109.78
	L	28.00	114.76	119.66	28.76	118.93
70-79	C	18.00	181.28	182.37	41.85	174.52
	T	18.00	128.79	130.00	30.94	127.86
	L	18.00	99.29	96.69	31.48	140.82
80-89	C	12.00	174.13	171.75	37.75	129.21
	T	12.00	125.55	118.38	29.52	92.80
	L	12.00	97.55	96.07	27.16	79.37
Total	C	200.00	228.67	228.67	54.62	331.99
	T	200.00	169.31	168.19	40.39	231.87
	L	200.00	149.69	147.76	44.96	269.18
Age group	N	**Mean (mg/cm**^**3**^**)**	Median (mg/cm^3^)	Standard deviation	Minimum	Maximum
10-19 years	17	225.69	221.68	37.37	171.90	337.50
20-29 years	25	208.64	200.59	27.51	169.17	280.06
30-39 years	36	209.26	206.09	34.68	131.48	281.82
40-49 years	25	199.90	197.68	29.95	154.84	261.39
50-59 years	39	162.51	156.71	34.88	116.56	305.27
60-69 years	28	156.66	151.13	31.78	111.17	220.64
70-79 years	18	136.35	138.85	33.32	69.89	217.62
80-89 years	12	128.73	138.68	30.07	86.59	186.77
Total	200	181.54	181.60	44.59	69.89	337.50

*N* number of cases, *C* Cervical, *T* Thoracic, *L* Lumbal

**Fig. 3:**
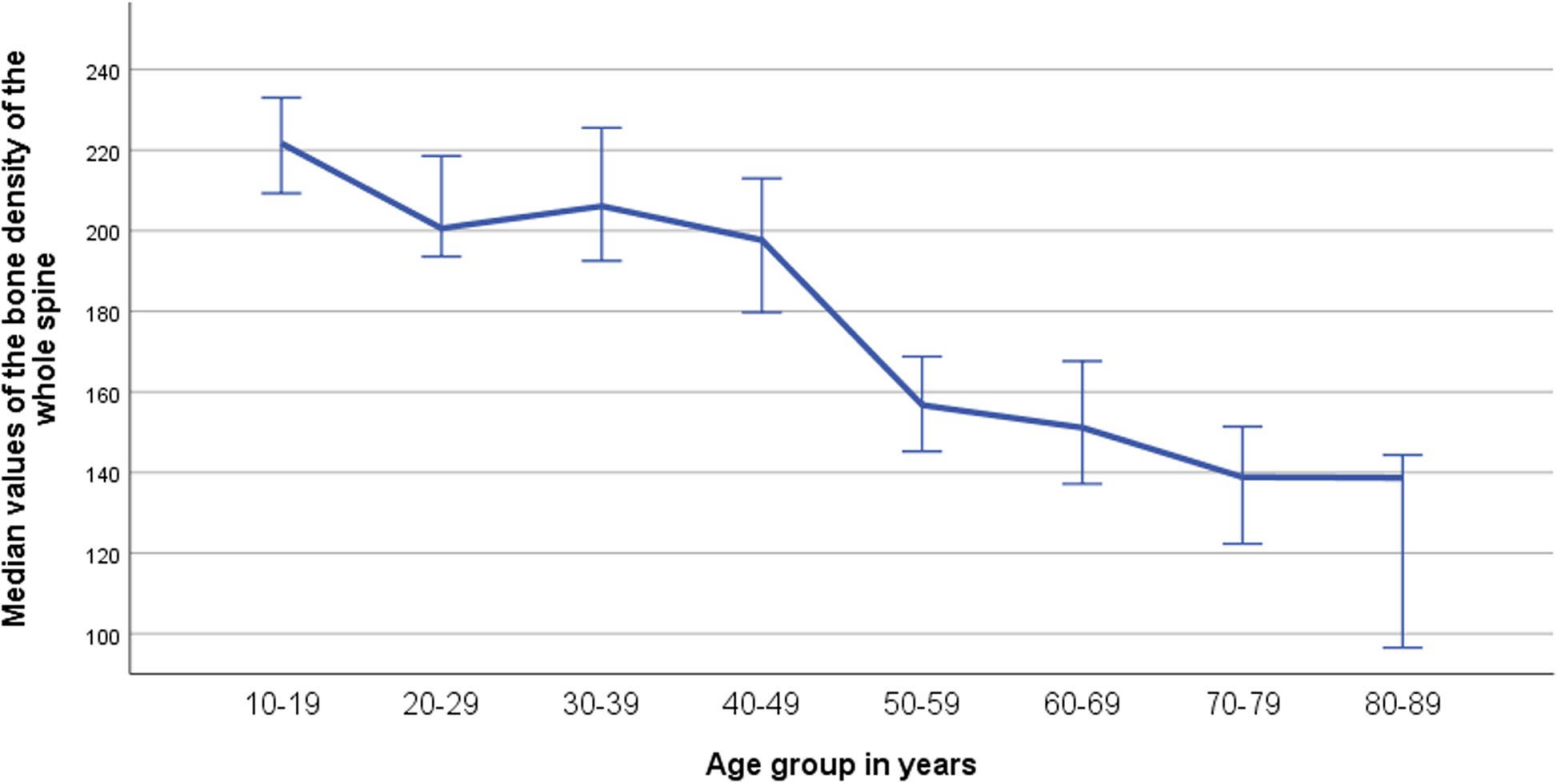
Diagram showing bone density values of the entire spine distributed among age groups. The error bars represent 95% confidence intervals

A reduction in bone density was observed in patients of 50 years of age and older, with a statistically significant difference in paired comparisons of age groups below 50 years with those of 50 years and above, when comparing the bone density of the whole spine (*p*<0.001) (Fig. 3).

**Fig. 4:**
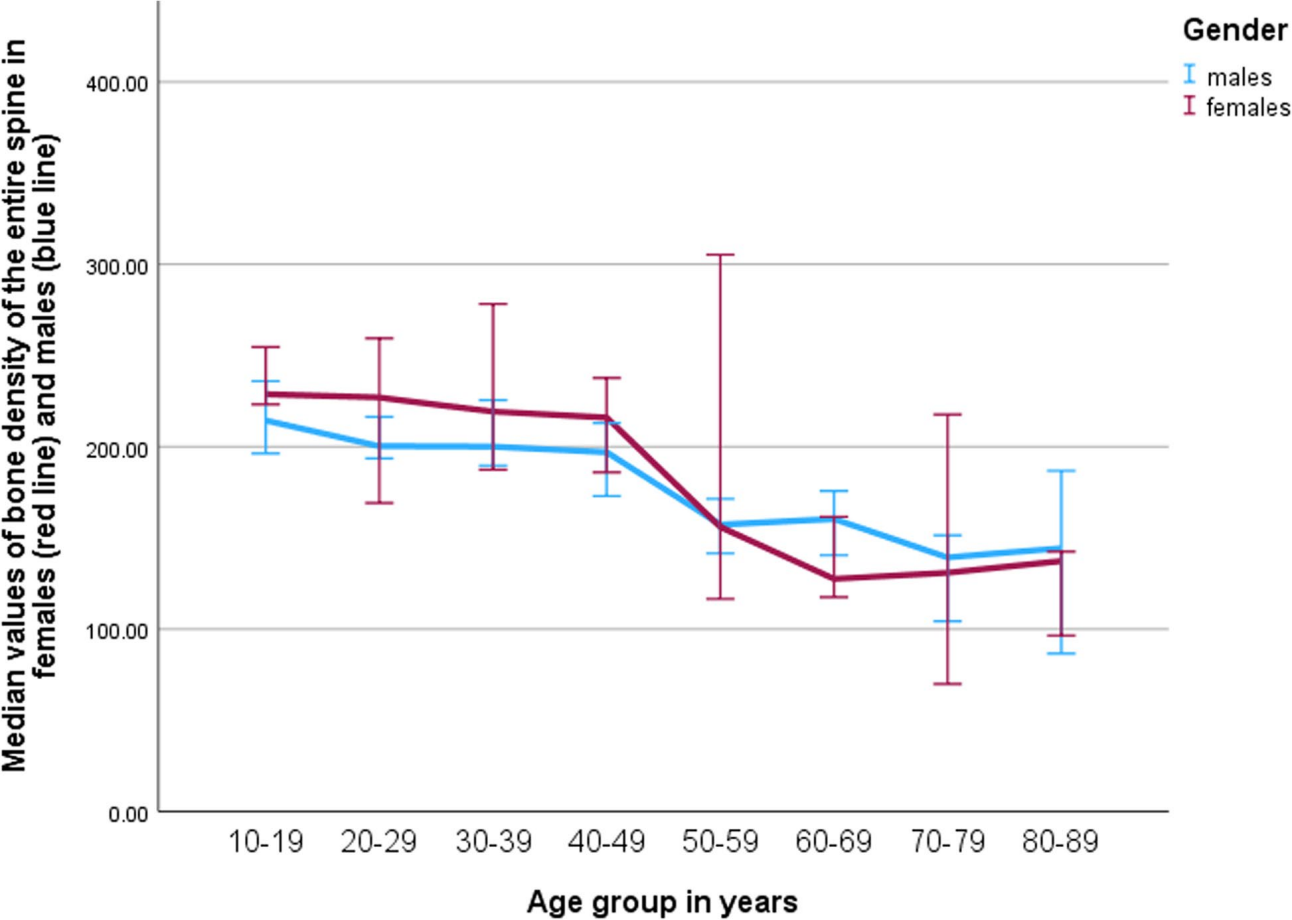
Diagram showing bone density values of the entire spine distributed among age groups in females and males. The error bars represent 95% confidence intervals

Similar results were seen when comparing bone density values of the age groups below 50 years with those of 50 years or above for the cervical, thoracic as well as lumbar spine. The decrease in values was observed in both females and males (Fig. 4). Almost all of the pairwise comparisons between each group under 50 years and each group over 50 years, for the different spine regions and sex-based comparisons, were found to be statistically significant in the analysis. The remaining comparisons showed a trend towards significance.

## Discussion

The defining features of osteoporosis include a decline in bone density and modifications to the basic structure of bone. Regular assessments of bone density can enable earlier diagnosis of osteoporosis, while bone density measurement is also a key factor to take into account when a spinal instrumentation is planned. There are a few studies in the literature describing the differences in bone density between different spinal segments. This data has implications not only for osteoporosis diagnosis and spine surgery preparation, but also for scientists and medical device companies interested in studying the biomechanical features of diverse vertebral bodies. The bone density of the cervical spine [27] or lumbar spine using HU [28] has been measured previously. To our knowledge, there is no study that has investigated the bone density of the entire spine. Weishaupt et al. published 2001 QCT data only of the vertebral bodies of C2, C5, T12, and L4 from 50 healthy volunteers [29].

Evidence suggests that the introduction of contrast agent into the body results in a increase in HU values and substantially affects BMD-assessment on CT, as previously documented in the literature [30]. On average, the difference between native and arterial phase in the study by Pompe et al. was 12 HU [31].

Our study focused on the measurement of bone density throughout the entire spine, and we consider it to be one of the principal objectives. A notable strength of our research is the considerable number of patients who were included in the study, all of whom underwent an identical examination, including the administration of contrast medium, to ensure consistency and comparability of values across patients. Bone density was measured in three different locations within the vertebral body: below the upper plate, in the middle of the vertebral body, and above the base plate. Compared to measuring only in one part of the vertebral body, we find this method more accurate and representative of the bone properties of whole vertebral body.

In our study, bone density decreased from cranial to caudal with higher values in the cervical spine. Similar results in the cervical spine as well as lumbar spine were described in the study by Zhang et al. In this study, bone density of the cervical spine and lumbar spine was measured by QCT in 598 patients. The density of the cervical spine was higher than the density of the lumbar spine.

In the study by Curylo et al. examining bone density in the lower cervical spine in cadavers, lower values were described in C7 compared with the other cervical vertebral bodies examined (C4-6) [32]. Anderst et al. and Weishaupt et al. reported that the measured bone density of the fifth cervical vertebra was greatest when assessing the cervical vertebrae from C3 to C7 [27, 29]. The higher values of bone density in the cervical spine may be explained by the greater loads and forces to which they are exposed due to their mobility and small size [28, 33] According to H.J. Grote et al. another explanation could be that the dense structure of cervical vertebrae is a phylogenetic reminiscence of quadruped gait, where the head-carrying cervical spine was exposed to much higher stress [33]. The data from our study in the cervical spine are generally consistent with those from the literature. In the lower cervical spine (C6 and C7), the values in our study were statistically significantly lower than the values in the upper cervical spine (*p*< 0.001).

The studies of Yoganandan et al. are the only one, to our knowledge, in which bone density in the thoracic spine was measured by QCT in addition to the cervical and lumbar spine [34, 35]. It is also worth mentioning that in these studies, the bone density of only one vertebral body of the thoracic spine was measured (T12). There was a decrease in bone density from cranial to caudal in this study as well. The study by Salzmann et al. examined bone density of the entire thoracic spine. The bone density of 50 male patients was examined by QCT. The bone density of all thoracic vertebral bodies cranial to T6 was statistically higher than the bone density of the vertebral bodies caudal to T6 [36].

In our study, the same decrease in bone density values from cranial to caudal was noted in the thoracic spine, with statistically higher values in the upper thoracic spine (T1-4) compared with the middle and low spine. An advantage of our study compared with Yoganandan's studies is that we measured bone density of all thoracic vertebral bodies. Another strength of our study is the significantly higher and more representative number of patients included.

In the lumbar spine, Zhang et al. described no relevant differences in bone density between the individual lumbar vertebral bodies [28]. Similarly, in the studies by Yoganandan, no relevant differences in bone density were described between the different lumbar vertebral bodies studied from L2 to L4 [34, 35]. In our study, the values of the vertebral body L1 and L5 were higher than the other values (*p*< 0.001). There was no significant difference between the bone density values of L2, L3 and L4. The difference could be attributed to a possible increased force load in the thoracolumbar and lumbosacral junction.

A few studies have investigated the differences in spinal bone density between males and females. Lehmann et al. reported no significant differences in bone density between premenopausal women and men [37]. These findings were consistent with similar results reported by Cvijetic and Korsic [38]. However, there are several studies describing higher bone density values for premenopausal women compared with men [5, 34]. Zhang et al. described greater values of bone density in women compared with men at all ages. All patients were under 65 years old [28].

When comparing bone density by gender, we did not find a significant statistical difference in values in our study. The bone density values of the cervical, thoracic, as well as lumbar spine by gender were similar, with no significant differences.

By analyzing bone density across different age groups, we observed that both men and women over the age of 50 exhibited lower total spine bone density in our study. This decrease in women's bone density can be attributed to postmenopausal changes, which is consistent with findings from Lehman et al. They used DXA for bone density measurement [37]. However, the same authors did not observe a significant decrease in bone density with the increase in age in men. In a study by Zhang et al., it was observed that women had higher bone density values compared to men across all age groups [28]. Differences in research methodologies may account for the disparities between the studies of Lehmann et al. and Zhang et al. It is known that DXA examination assesses both cortical and trabecular bone. The observation of a decrease in bone density among postmenopausal women is highly credible from a physiological standpoint. Our study similarly confirmed this finding. The substantial decline in bone density among men older than 50 years could be attributed to a decrease in testosterone levels. Nevertheless, it is widely acknowledged that testosterone levels typically decrease by only around 1 percent each year [39]. Another possible explanation is that bones may be subjected to less stress due to reduced physical activity as people age, leading to a decline in bone density. Several randomized studies have already demonstrated the beneficial impact of strength training on bone density [40, 41].

It is important to consider the limitations of our study, which include its retrospective and monocentric nature. Our study used CT scans that were conducted in the context of trauma, which may have led to inclusion of patients with pre-existing conditions. In addition, the study did not collect data on medications that may impact bone density or individual radiation dose values.

All patients underwent the examination with the administration of contrast medium, which should theoretically enhance the consistency and comparability of the results. However, it's important to note that the use of contrast medium can influence the measurement of bone density. Research by Islamian et al. suggests that CT-derived bone density values might be impacted by intravenous contrast medium, with this effect diminishing as patient age increases [30]. This presents a potential limitation, particularly in our age-related comparisons between different patients.

A limitation of this study is the fact that there were three times as many male patients as female patients, which is likely due to a higher prevalence of traumatic injury in men.

Another limitation of the study is the method used to determine bone density, which involves directly measured HU on CT using the equation of Buenger et al. [1]. Unlike other clinically used methods, this approach does not involve calibration of the bone density values and is thus dependent on the CT scanner used, which may result in differences in HU values obtained between CT scanners from different manufacturers. This was confirmed by a study of 67392 CT investigations obtained from four different CT scanners [23].

In order to reduce the potential for errors in the measurement of HU on CT scans from different scanners, we ensured that all scans in our study were obtained using the same CT scanner.

It is worth mentioning that we did not assess inter-observer reliability in our study, which could impact the precision of the HU measurements. All HU measurements of the spine were carried out by a single investigator. Nonetheless, a recent study conducted at our institution in 2022 showed that the inter-observer reliability of direct HU measurement on the same vertebra was excellent among four different investigators [42].

Another potential limitation is the possibility of selection bias resulting from the exclusion criteria. The average bone density may be artificially inflated because patients with very low bone density may have been excluded too frequently, particularly those with pathology in more than three vertebrae.

It's worth noting that selection bias due to the exclusion criteria could be a limiting factor in this study. The exclusion of patients with pathology in more than three vertebrae may have resulted in an overrepresentation of patients with higher bone density, potentially inflating the average value.

## Conclusion

The bone density values of the cervical, thoracic, and lumbar spine in 200 patients were assessed in this study using Hounsfield units (HU).

The average spinal bone density values decreased from cranial to caudal (*p* < 0.001). The highest values were seen in the upper cervical spine.

The study's findings showed that bone density values determined by HU measurements generally decreased with age in all spinal segments. Notably, there was a marked reduction in bone density in both males and females after the age of 50.

The information obtained on bone density in specific spinal segments may prove valuable in the preoperative assessment of the spine, as well as in the design of medical instruments such as screws and disc replacements. The bone density measurements can guide the adaptation of these devices to the varying anatomical and biomechanical demands of different spinal regions.

## Data Availability

The data that support the findings of this study are available on request from corresponding author, George Simion. The data are not publicly available because of their containing information that could compromise the privacy of the patients.
